# Anaemia and pathologic complete response rate according to carboplatin dose in HER2+ breast cancer treated with neoadjuvant TCHP

**DOI:** 10.1002/cam4.5022

**Published:** 2022-07-15

**Authors:** Jung Hwan Ji, Soong June Bae, Seul‐Gi Kim, Min Hwan Kim, Gun‐Min Kim, Joohyuk Sohn, Joon Jeong, Jee Hung Kim, Sung Gwe Ahn

**Affiliations:** ^1^ Department of Surgery, Gangnam Severance Hospital Yonsei University College of Medicine Seoul Republic of Korea; ^2^ Institute for Breast Cancer Precision Medicine Yonsei University College of Medicine Seoul Republic of Korea; ^3^ Division of Medical Oncology, Department of Internal Medicine Yonsei University College of Medicine Seoul Republic of Korea; ^4^ Division of Medical Oncology, Department of Internal Medicine, Gangnam Severance Hospital Yonsei University College of Medicine Seoul Republic of Korea

**Keywords:** anaemia, breast cancer, carboplatin, neoadjuvant chemotherapy, pathologic complete response

## Abstract

Grade 3/4 anaemia, which is mainly induced by carboplatin, frequently occurs in patients treated with neoadjuvant docetaxel/carboplatin/trastuzumab/pertuzumab (TCHP). However, dose reduction of carboplatin may raise concerns about the oncological outcome. This study investigated the pathologic complete response (pCR) rate, occurrence of grade 3/4 anaemia, and transfusion rate according to carboplatin dose in patients treated with neoadjuvant TCHP. We retrospectively analysed 294 patients treated with neoadjuvant TCHP between April 2015 and December 2020. Case matching was performed using propensity score matching. Among patients treated with neoadjuvant TCHP, carboplatin area under the plasma concentration–time curve 6 (AUC6) was used in 234 patients (79.6%) and upfront carboplatin AUC5 was used in 60 patients (20.4%). No significant difference in pCR rate was found between the two groups (AUC6: 70.9%, AUC5: 80.0%). In both oestrogen receptor‐positive (ER+) and ER‐ patients, no significant differences were observed between the AUC6 and AUC5 groups (ER+: 54.3% vs. 50.0%, ER‐: 81.7% vs. 86.0%). The case‐matched cohort showed consistent findings. The AUC5 group had lower frequencies of grade 3/4 anaemia (18.3% vs. 34.2%) and transfusion events (10.0% vs. 21.8%) than the AUC6 group. Compared with AUC5, carboplatin at AUC6 would associate with a 2.7‐fold increased risk of grade 3 or 4 chemotherapy‐induced anaemia. Carboplatin AUC5 has comparable cytotoxic effects to carboplatin AUC6 in patients with HER2+ breast cancer treated with six cycles of neoadjuvant TCHP, with fewer complications associated with clinically meaningful anaemia. AUC5 may be the optimal carboplatin dose to reduce TCHP‐induced anaemia in patients with HER2+ breast cancer treated with TCHP.

## Graphical abstract

Carboplatin AUC5 can provide a comparable cytotoxic effect to carboplatin AUC6 in terms of pathologic complete response in patients with HER2+ breast cancer receiving neoadjuvant docetaxel/carboplatin/trastuzumab/pertuzumab (TCHP), with fewer complications associated with grade 3/4 anemia. We suggest that AUC5 can be considered the optimal dose of carboplatin for patients with HER2+ breast cancer treated with TCHP.

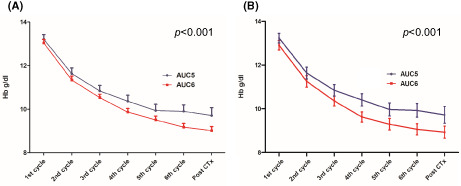

## NOVELTY & IMPACT STATEMENT

Carboplatin AUC5 can provide a comparable cytotoxic effect to carboplatin AUC6 in terms of pathologic complete response in patients with HER2+ breast cancer receiving neoadjuvant docetaxel/carboplatin/trastuzumab/pertuzumab (TCHP), with fewer complications associated with grade 3/4 anaemia. We suggest that AUC5 can be considered the optimal dose of carboplatin for patients with HER2+ breast cancer treated with TCHP.

## 1 BACKGROUND

Human epidermal growth factor receptor 2 (HER2)‐positive breast cancer accounts for approximately 15–20% of all breast cancers.^1^ Since the approval of trastuzumab, a HER2‐targeted monoclonal antibody, in the United States in 1998, many HER2‐targeted treatments have been developed. HER2‐targeted therapy has greatly contributed to the improvement of the survival rate of patients with breast cancer.^2^ In the neoadjuvant setting, the use of a trastuzumab‐based regimen for HER2+ breast cancer has resulted in a high pathologic complete response (pCR) rate, which indicates a good patient prognosis.^3^, ^4^, ^5^ Furthermore, pertuzumab, a new class of drug that inhibits HER dimerisation, has been incorporated into neoadjuvant treatments.^6^, ^7^ Thereafter, a dual HER2 blockade regimen consisting of docetaxel/carboplatin/trastuzumab/pertuzumab (TCHP) achieved high pCR rates of 55.7–66.2%.^8^, ^9^ On the basis of these results, a regimen consisting of six cycles of TCHP has been the most preferred neoadjuvant treatment for HER2+ breast cancer with tumour size >2 cm or with lymph node metastasis.^10^

However, the TCHP regimen includes two cytotoxic drugs (docetaxel and carboplatin) with many known adverse effects. Particularly, carboplatin causes haematological adverse events such as anaemia and thrombocytopenia.^11^, ^12^ Thus, in patients receiving neoadjuvant TCHP, anaemia and thrombocytopenia are adverse effects of special interest. Notably, in previous studies, grade 3/4 anaemia developed in >10% of patients treated with TCHP (10–17.1%).^8^, ^9^, ^13^, ^14^, ^15^ Furthermore, recent real‐world data showed that approximately 20% of patients treated with neoadjuvant TCHP needed blood transfusion.^15^ As anaemia and anaemia‐related adverse events can delay chemotherapy or surgery, which may affect the treatment results,^16^, ^17^ a proactive approach against carboplatin‐induced anaemia is clinically important in patients receiving TCHP. Nevertheless, none of the guidelines provide special considerations with respect to preventing or managing anaemia associated with the TCHP regimen.^18^, ^19^ Conversely, dose reduction of carboplatin may raise concerns about the oncological outcome of the treatment.

In this study, we compared the pCR rates according to carboplatin dose in patients with HER2+ breast cancer treated with neoadjuvant TCHP. Moreover, we investigated the occurrence of grade 3/4 anaemia and the blood transfusion rate according to the carboplatin dose and identified risk factors for grade 3/4 anaemia.

## 2 METHODS

### 2.1 Patients

We retrospectively collected the data of patients with non‐metastatic, HER2+ breast cancer treated with neoadjuvant TCHP at Gangnam and Sinchon Severance Hospital between 1 April 2015 and 31 December 2020. All patients had histologically confirmed HER2+ invasive breast carcinoma and underwent curative surgery after neoadjuvant TCHP treatment. Information about clinical and pathological characteristics, including body mass index, were collected through a review of electronic medical records. The HER2 status was defined based on immunohistochemical analysis. Patients with a score of 3+ were considered HER2+, and those with a score of 0 or 1 were considered HER2–.^20^ In patients with a score of 2+, tumour tissues were subjected to fluorescence in situ hybridisation assay using the PathVysion kit (Vysis, Downers Grove, IL, USA) or INFORM HER2 (Ventana, Tucson, AZ, USA), according to the manufacturer's instructions.^1^ The clinical stages were determined according to the seventh edition of the American Joint Committee on Cancer guidelines.^21^

The study protocol was approved by the institutional review board of Gangnam and Sinchon Severance Hospital, Yonsei University, Seoul, Republic of Korea. Owing to the retrospective study design, the need for informed consent was waived by the institutional review board.

### 2.2 TCHP treatment and carboplatin dose

Drugs were intravenously administered on two consecutive days (trastuzumab followed by pertuzumab on day 1 and carboplatin followed by docetaxel on day 2) every 3 weeks for six cycles. Trastuzumab was administered at 8 mg/kg on cycle 1 and at 6 mg/kg thereafter. Pertuzumab was administered at 840 mg on cycle 1 and at 420 mg thereafter. Carboplatin was initially administered at a dose of area under the plasma concentration–time curve 5 or 6 (AUC5 or AUC6), and docetaxel was administered at 75 mg/m^2^. Carboplatin dose was calculated using the Calvert formula.^22^ The maximum dose was based on an estimated glomerular filtration rate capped at 125 ml/min for patients with normal renal function.^23^ The maximum doses were calculated as follows:

Maximum carboplatin dose (mg) = target AUC (mg/mL/min) × (125 ml/min + 25).

For a target AUC of 6, the maximum dose was 6 × 150 = 900 mg.

For a target AUC of 5, the maximum dose was 5 × 150 = 750 mg.

The carboplatin dose was determined by the physician considering the patient's general characteristics, such as age and underlying diseases.

### 2.3 Assessment of outcomes and anaemia

We compared the pCR rates according to the carboplatin dose. pCR was defined as the absence of invasive tumour cells in both the breast and axillary lymph nodes (ypT0/Tis, ypN0) based on the pathological evaluation of surgical specimens. Serial haemoglobin (Hb) and platelet (Plt) levels were investigated by measuring the Hb levels at baseline, before each chemotherapy cycle, and after chemotherapy completion. The grade of anaemia was assessed in accordance with the National Cancer Institute Common Terminology Criteria for Adverse Events version 4. Transfusions of packed red blood cells during the neoadjuvant treatment was investigated.

### 2.4 Statistical analysis

Continuous variables were compared using Student's *t*‐test. Categorical variables were compared using the χ^2^ or Fisher's exact tests. To compare serial Hb levels as a continuous variable according to carboplatin dose, a two‐way analysis of variance test was applied. To reduce selection bias between the two groups according to carboplatin dose, we performed a 1:2 propensity score matching (PSM) analysis. The matching covariates were age and oestrogen receptor (ER) status. Nearest‐neighbour matching without replacement was used to perform matching, and the calliper was set to 0.25. The PSM procedure was performed using the *MatchIt* R package.

To identify predictive factors of grade 3/4 anaemia and transfusion events, multivariable binary logistic regression analysis was performed. The stepwise‐backward Wald method was used to obtain the final model. Odds ratios and 95% confidence intervals with two‐sided *p*‐values were calculated. All statistical analyses were performed using R software (https://www.r‐proget.org; version 4.1.1) and SPSS (version 25.0; SPSS Inc., Chicago, IL, USA). Statistical significance was set at *p* < 0.05.

## 3 RESULTS

### 3.1 Patient demographics

The schematic diagram of our study is provided in Figure S1. All patients completed six cycles of neoadjuvant TCHP except for three patients (one patient received five cycles of TCHP before surgery and other the two patients received three cycles of TCHP). Among a total of 294 patients treated with neoadjuvant TCHP, carboplatin AUC6 was used in 234 patients (79.6%, 234 of 294) and upfront carboplatin AUC5 was used in 60 patients (20.4%, 60 of 294). We compared the clinical and pathological features according to the carboplatin dose (Table 1). The median age in the AUC5 group was higher than that in the AUC6 group (56.5 vs. 49 years, *p* = 0.001). Furthermore, the AUC5 group had a higher proportion of ER‐ patients than the AUC6 group (83.3% vs. 60.7%, *p* = 0.001; Table 1). To minimise the baseline confounders between the two groups, 1:2 PSM was performed. After the PSM procedure, the baseline characteristics of 168 patients (112 patients in the AUC6 group and 56 patients in the AUC5 group) were well balanced (Table 1).

**TABLE 1:** Baseline characteristics according to carboplatin dose

Variables	Whole cohort				Matched cohort			
	AUC6 (*n* = 234)	AUC5 (*n* = 60)	Total (*N* = 294)	*p*‐value	AUC6 (*n* = 112)	AUC5 (*n* = 56)	Total (*n* = 168)	*p*‐value
Age, years (median, range)	49 (27–75)	56.5 (31–69)	50 (27–75)	0.001	54 (31–72)	54 (31–72)	54 (31–72)	0.572
cT stage				0.381^a^				0.121^a^
1	12 (5.1%)	5 (8.3%)	17 (5.8%)		5 (4.5%)	5 (8.9%)	10 (6.0%)	
2	153 (65.4%)	42 (70.0%)	195 (66.3%)		70 (62.5%)	40 (71.4%)	110 (65.5%)	
3	69 (29.5%)	13 (21.7%)	82 (27.9%)		37 (33.0%)	11 (19.6%)	48 (28.6%)	
cN stage				0.533^a^				0.102^a^
0	47 (20.1%)	13 (21.7%)	60 (20.4%)		19 (17.0%)	12 (21.4%)	31 (18.5%)	
1	122 (52.1%)	33 (55.0%)	155 (52.7%)		55 (49.1%)	30 (53.6%)	85 (50.5%)	
2	59 (25.2%)	11 (18.3%)	70 (23.8%)		37 (33.0%)	11 (19.6%)	48 (28.6%)	
3	6 (2.6%)	3 (5.0%)	9 (3.1%)		1 (0.9%)	3 (5.4%)	4 (2.4%)	
Oestrogen receptor status				0.001				0.77
Negative	142 (60.7%)	50 (83.3%)	192 (65.3%)		94 (83.9%)	46 (82.1%)	141 (83.3%)	
Positive	92 (39.3%)	10 (16.7%)	102 (34.7%)		18 (16.1%)	10 (17.9%)	28 (16.7%)	
BMI, kg/m^2^				0.765				0.804
<25	176 (75.2%)	44 (73.3%)	220 (74.8%)		82 (73.2%)	42 (75.0%)	124 (73.8%)	
≥25	58 (24.8%)	16 (26.7%)	74 (25.2%)		30 (26.8%)	14 (25.0%)	44 (26.2%)	

*Note*: Abbreviations: AUC, area under the plasma concentration–time curve; BMI, body mass index.

a Fisher's exact test.

### 3.2 pCR rate according to carboplatin dose

**FIGURE 1:**
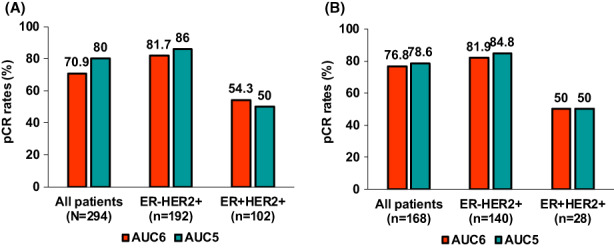
Pathological complete response (pCR) rates according to carboplatin dose. (A) pCR rates according to carboplatin dose in the whole cohort (*N* = 294). pCR status according to carboplatin dose was compared using Fisher's exact tests in all patients, in a subset of patients with ER‐HER2+ disease, and in a subset of patients with ER + HER2+ disease (*p* = 0.159, *p* = 0.487, and *p* = 0.793, respectively). (B) pCR rates according to carboplatin dose in the matched cohort (*n* = 168). pCR status according to carboplatin dose was compared using Fisher's exact tests in all patients, in the ER‐HER2+ subgroup, and in the ER + HER2+ subgroup (*p* = 0.794, *p* = 0.672, and *p* > 0.999, respectively).

In the whole cohort, the pCR rate was 72.8%. The pCR rate did not differ according to carboplatin dose. pCR was achieved in 70.9% (166 of 234) of patients in the AUC6 group and in 80.0% (48 of 60) of patients in the AUC5 group (*p* = 0.159; Figure 1A). No difference in pCR rates according to carboplatin dose was found in both the ER + HER2+ and ER‐HER2+ subgroups (Figure 1A). In patients with ER + HER2+ disease, the pCR rate was 54.3% (50 of 92) in the AUC6 group and 50% (5 of 10) in the AUC5 group. In those with ER‐HER2+ disease, the pCR rates were 81.7% (116 of 142) and 86.0% (43 of 50) in the AUC6 and AUC5 groups, respectively (Figure 1A).

Consistent findings were obtained in the PSM cohort, which had a pCR rate of 77.4%. The pCR rate did not significantly differ according to the carboplatin dose (76.8% in the AUC6 group and 78.6% in the AUC5 group; Figure 1B). Additionally, no differences in pCR rates were found according to carboplatin dose in both the ER + HER2+ and ER‐HER2+ subgroups (Figure 1B).

### 3.3 Grade 3/4 anaemia, transfusion, and risk factors

**FIGURE 2:**
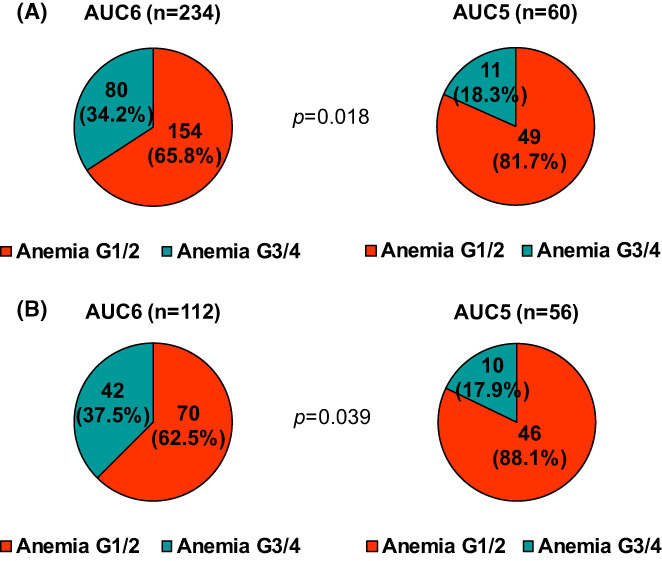
Distribution of anaemia grade according to carboplatin dose. The proportions of grade 3/4 anaemia according to carboplatin dose were compared using Fisher's exact tests in the (A) whole cohort (*N* = 294) and (B) matched cohort (*n* = 168) (*p* = 0.018 and *p =* 0.039, respectively).

**FIGURE 3:**
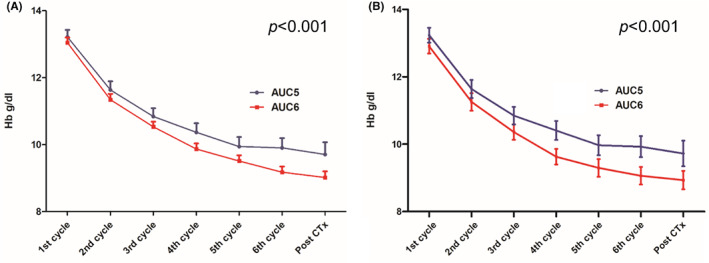
Serial haemoglobin (Hb) levels during neoadjuvant docetaxel/carboplatin/trastuzumab/pertuzumab treatment according to carboplatin dose. Serial Hb levels according to carboplatin dose were compared using two‐way analysis of variance tests in the (A) whole cohort (*N* = 294) and (B) matched cohort (*n* = 168) (*p* < 0.001 and *p <* 0.001, respectively).

We investigated the occurrence of grade 3/4 anaemia and the transfusion rates. The nadir grade of anaemia during the treatment was evaluated. All patients developed at least grade 1 anaemia during TCHP treatment. Grade 3/4 anaemia was observed in 31.0% (91 of 294) of patients. It was more frequently observed in patients treated with carboplatin AUC6 in both the whole and case‐matched cohorts (*p* = 0.018 and *p* = 0.009, respectively; Figure 2). Approximately 35% of patients in the AUC6 group developed grade 3/4 anaemia, whereas <20% of patients in the AUC5 group had grade 3/4 anaemia. Using two‐way analysis of variance tests, we compared Hb levels serially measured during the neoadjuvant treatment according to carboplatin dose. These analyses indicated that carboplatin dose was a significant factor for a steeper decline in the serial Hb levels observed in the AUC6 group (Figure 3). The results were similar between the two cohorts. Additionally, when we compared the serial Plt counts between two groups, the level of Plt decreased sharply in the AUC6 than in the AUC5 in the whole patients (*p* = 0.010; Figure S2). However, it was not significant in the matched cohort despite a trend (*p* = 0.283).

**FIGURE 4:**
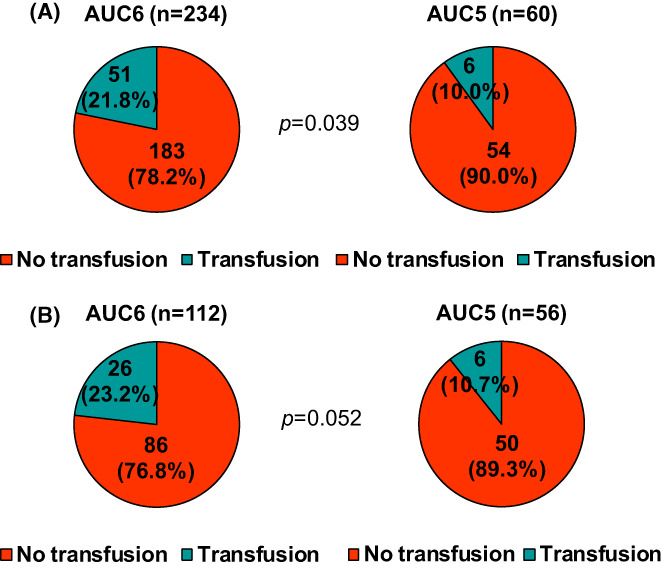
Red blood cell (RBC) transfusion rates according to carboplatin dose. The rates of RBC transfusion according to carboplatin dose were compared using Fisher's exact tests in the (A) whole cohort (*N* = 294) and (B) matched cohort (*n* = 168) (*p* = 0.039 and *p =* 0.052, respectively).

Of the total patients, 57 (19.4%) received red blood cell transfusion during preoperative TCHP treatment. The transfusion rate was higher in the AUC6 group (21.8%) than in the AUC5 group (10.0%) in the whole cohort (*p* = 0.039; Figure 4). Similarly, the transfusion rate tended to be higher in the AUC6 group (23.2%) than in the AUC5 group (10.7%) in the case‐matched cohort (*p* = 0.052; Figure 4).

In addition, we investigated other risk factors associated with grade 3/4 anaemia, including carboplatin dose (Table 2). In univariable analyses, low baseline Hb level (<12 g/dl), a rapid drop in Hb level of ≥2 g/dl (Hb drop >2 g/dl after the first cycle), and carboplatin dose were significantly associated with grade 3/4 anaemia. Multivariable analysis revealed baseline Hb <12 g/dl and a rapid drop in Hb level as independent variables associated with grade 3/4 anaemia (Table 2). In this analysis, carboplatin dose was a marginally significant factor (odds ratio, 2.06; 95% confidence interval, 0.99–4.29; *p* = 0.053). However, in the case‐matched cohort, carboplatin dose was significantly associated with grade 3/4 anaemia (odds ratio, 2.68; 95% confidence interval, 1.18–6.10; *p* = 0.019), in addition to low baseline Hb level and a rapid drop in Hb level after the first cycle. Within the carboplatin AUC5 group (*n* = 60), low baseline Hb level was significantly associated with grade 3/4 anaemia in univariable analysis (Table S1). Owing to the small number of patients, multivariable analysis was not performed within this group. When we compared the baseline Hb levels according to the carboplatin dose, no statistically significant differences were observed between the two groups in both cohorts (Table S2).

**TABLE 2:** Univariable and multivariable binary logistic regression analyses to identify risk factors for grade 3/4 anaemia

Variables	Whole cohort				Matched cohort			
	Univariable		Multivariable		Univariable		Multivariable	
	Odds ratio (95% CI)	*p*‐value	Odds ratio (95% CI)	*p*‐value	Odds ratio (95% CI)	*p*‐value	Odds ratio (95% CI)	*p*‐value
Baseline Hb, g/dl		0.034		0.013		0.071		0.031
≥12	Ref.		Ref.		Ref.		Ref.	
<12	2.19 (1.06–4.53)		2.67 (1.23–5.76)		2.49 (0.93–6.69)		3.24 (1.11–9.46)	
Hb change^a^, g/dl		<0.001		<0.001		<0.001		<0.001
<2	Ref.		Ref.		Ref.		Ref.	
≥2	3.27 (1.95–5.47)		3.45 (2.02–5.87)		3.67 (1.84–7.32)		4.05 (1.95–8.39)	
AUC		0.02		0.053		0.011		0.019
5	Ref.		Ref.		Ref.		Ref.	
6	2.31 (1.14–4.70)		2.06 (0.99–4.29)		2.76 (1.26–6.04)		2.68 (1.18–6.10)	
BMI, kg/m^2^		0.75				0.6		
<25	Ref.				Ref.			
≥25	1.10 (0.62–1.93)				1.22 (0.59–2.53)			

*Note*: Abbreviations: AUC, area under the plasma concentration–time curve; BMI, body mass index; CI, confidence interval; Hb, haemoglobin; Ref., reference.

a Rapid drop in haemoglobin level after the first cycle.

## 4 DISCUSSION

In this study, carboplatin AUC5 did not decrease the pCR rate in patients with HER2+ breast cancer who received neoadjuvant TCHP, eliminating the concerns about poorer oncological outcome, in terms of pCR, related to carboplatin dose reduction. Moreover, carboplatin AUC5 had an advantage over carboplatin AUC6 in reducing grade 3/4 anaemia, suggesting that the use of a reduced dose enables avoiding transfusion requirements and treatment delays. Lastly, we found that baseline anaemia and a rapid drop in the Hb level are risk factors for severe anaemia during treatment.

A previous study of 124 patients showed that carboplatin dose capping resulted in inferior pCR rates in the ER + HER2+ subgroup, suggesting that a suboptimal dose of carboplatin potentially hampers the achievement of the expected oncological outcome in patients treated with TCHP.^24^ Conversely, our data showed that TCHP with carboplatin AUC5 offered non‐inferior pCR rates to carboplatin AUC6 in approximately 300 patients. Our findings suggest that carboplatin AUC5 can provide an equivalent cytotoxic effect to carboplatin AUC6 in patients with HER2+ breast cancer receiving six cycles of neoadjuvant treatment consisting of TCHP, with fewer complications associated with clinically significant anaemia.

In the earlier era of carboplatin use, platinum compounds, including carboplatin, have been consistently associated with clinically significant anaemia, resulting in the need for transfusion in 20–40% of patients.^25^, ^26^, ^27^ The mechanisms of platinum‐induced anaemia involve direct suppression of erythroid progenitor cells within the bone marrow and nephrotoxic effects on erythropoietin‐producing cells within the kidney.^28^, ^29^, ^30^ In addition, the myelotoxic impact of platinum salts manifests in a dose‐dependent manner, indicating that severe anaemia increases in proportion to increasing dose, increasing number of cycles, and decreasing time interval between treatment cycles.^31^ Our findings are consistent with previous data showing that grade 3/4 anaemia was more frequent and the transfusion rate tended to be higher in patients treated with carboplatin AUC6 than in those treated with carboplatin AUC5.

Factors such as baseline Hb level, body mass index, body surface area, advanced age, glomerular filtration rate, chemotherapy regimen, and female sex are generally considered important predictors of chemotherapy‐induced anaemia.^32^, ^33^ In addition, earlier studies suggested low baseline Hb level, early decrease in Hb level after treatment, cumulative platinum dose, advanced age, lack of chemotherapeutic response, and high residual platinum levels after administration as risk factors for platinum‐associated anaemia.^31^, ^34^ In this study, we identified baseline anaemia (Hb level < 12 g/dl), early drop in Hb level (a drop of >2 g/dl after the first cycle), and carboplatin dose (AUC6) as risk factors for grade 3/4 anaemia.

To prevent chemotherapy‐induced anaemia, iron supplementation combined with erythropoiesis‐stimulating agents has been proposed.^35^, ^36^, ^37^ Another study demonstrated that intravenous iron supplementation improved the Hb levels without erythropoiesis‐stimulating agents in patients undergoing cancer treatment.^38^ However, definitive guidelines for the use of iron therapy to reduce chemotherapy‐related anaemia are lacking. A paucity of data exists with respect to optimal iron supplementation strategies, long‐term safety, and impact of iron supplements on tumour growth. Our findings suggest that the occurrence of chemotherapy‐induced anaemia in patients receiving TCHP can be reduced by applying upfront dose reduction to carboplatin AUC5. As grade 3/4 anaemia developed in patients with baseline anaemia despite treatment with carboplatin AUC5, active iron supplementation should be considered in patients with risk factors. However, further evidence and prospective studies are required to confirm the usefulness of iron supplementation in patients with risk factors for chemotherapy‐induced anaemia.

The retrospective study design is the major limitation of this study. In addition, we briefly investigated thrombocytopenia in relation to carboplatin‐dose because we primarily aimed to evaluate an effect of carboplatin dose on Hb level. Lastly, we compared the oncological outcome only based on the pCR rates. Since our cohort has a short follow‐up duration (median 23 months, range 5–72), survival analyses can further confirm the non‐inferiority of TCHP with carboplatin AUC5 compared to TCHP with carboplatin AUC6 especially in the patients with non‐pCR.

In conclusion, carboplatin AUC5 can provide a comparable cytotoxic effect to carboplatin AUC6 in patients with HER2+ breast cancer receiving six cycles of neoadjuvant TCHP, with fewer complications associated with clinically meaningful anaemia. Baseline Hb levels <12 g/dl or a rapid Hb drop after the first cycle are risk factors for TCHP‐induced anaemia. Future studies should develop preventive strategies against TCHP‐induced anaemia for patients with these risk factors.

## AUTHORS' CONTRIBUTION

Study concept and design: J.H.J., S.K., J.S., J.J., J.H.K., S.G.A. Data acquisition, analysis, and interpretation: J.H.J., S.J.B., S.K., M.H.K., G.K., J.S., J.J., J.H.K., S.G.A. Statistical analysis: J.H.J., S.J.B., M.H.K., J.H.K., S.G.A. Drafting the manuscript: J.H.J., S.J.B., G.K., J.S., J.J., J.H.K., S.G.A. First author: J.H.J. Co‐corresponding author: J.H.K. and S.G.A. All authors reviewed and approved the final draft of the manuscript.

## FUNDING INFORMATION

This work was supported by funds from the Basic Science Research Program through the NRF (NRF‐2019R1C1C1002830), Republic of Korea.

## CONFLICT OF INTEREST

None of the co‐authors have any potential competing interests.

## ETHICS APPROVAL AND CONSENT TO PARTICIPATE

Retrospective cohort study design with ethical approval for retrospective analysis of human data was obtained under the Institutional Review Board of Gangnam Severance Hospital approval and informed consent was waived. The study was performed in accordance with the ethical standards of the institutional and/or national research committee and with the principles of the 1964 Declaration of Helsinki and its later amendments or comparable ethical standards.

## CONSENT FOR PUBLICATION

This manuscript does not contain any individual patient data.

## Supporting information


Appendix S1

## Data Availability

The data that support the findings of this work are available from the corresponding author upon request.
