# Supplementary material for: Anaemia and pathologic complete response rate according to carboplatin dose in HER2+ breast cancer treated with neoadjuvant TCHP
Source: Cancer Med. 2022 Jul 15;12(2):1409–17. doi: 10.1002/cam4.5022 (PMC9883435; doi:10.1002/cam4.5022)

Supplementary Materials

Chemotherapy-induced anemia and pathologic complete response rate according to carboplatin dose in HER2+ breast cancer treated with neoadjuvant TCHP

Jung Hwan Ji, Soong June Bae, Seul-Gi Kim, Min Hwan Kim, Gun-Min Kim, Joohyuk Sohn, Joon Jeong, Jee Hung Kim†, Sung Gwe Ahn†

\

†Co-corresponding authors: OK8504@yuhs.ac (Jee Hung Kim, ORCID: 0000-0002-9044-8540); asg2004@yuhs.ac (Sung Gwe Ahn, ORCID: 0000-0002-8778-9686); Tel.: 82-2-2019-3379

This file contains:

Supplementary Table 1

Supplementary Table 2

Supplementary Figure 1

Supplementary Figure 2

Supplementary Table 1. Univariable binary logistic regression analyses to identify risk factors for grade 3/4 anemia in the carboplatin AUC5 group (n=60)

Variables	Odds ratio (95% CI)	*p*-value
Baseline Hb, g/dL		0.028
≥12	Ref.	
<12	8.812 (1.27–61.32)	
Hb change*, g/dL		0.172
<2	Ref.	
≥2	2.57 (0.66–9.95)	
BMI, kg/m^2		0.425
<25	Ref.	
≥25	1.76 (0.44–7.08)	

*Rapid drop in hemoglobin level after the first cycle.

AUC, area under the plasma concentration–time curve; CI, confidence interval; Hb, hemoglobin; BMI, body mass index; Ref., reference

Supplementary Table 2. Baseline characteristics according to carboplatin dose

Variables	Whole cohort				Matched cohort			
	AUC6 (n=234)	AUC5 (n=60)	Total (N=294)	*p*-value	AUC6 (n=112)	AUC5 (n=56)	Total (n=168)	*p*-value
Hb change, g/dL				0.13				0.355
<2	143 (61.1%)	43 (71.7%)	186 (63.3%)		72 (64.3%)	40 (71.4%)	112 (66.7%)	
≥2	91 (38.9%)	17 (28.3%)	108 (36.7%)		40 (35.7%)	16 (28.6%)	56 (33.3%)	

AUC, area under the plasma concentration–time curve; Hb, hemoglobin

Supplementary Fig. 1 The schematic diagram of our study.

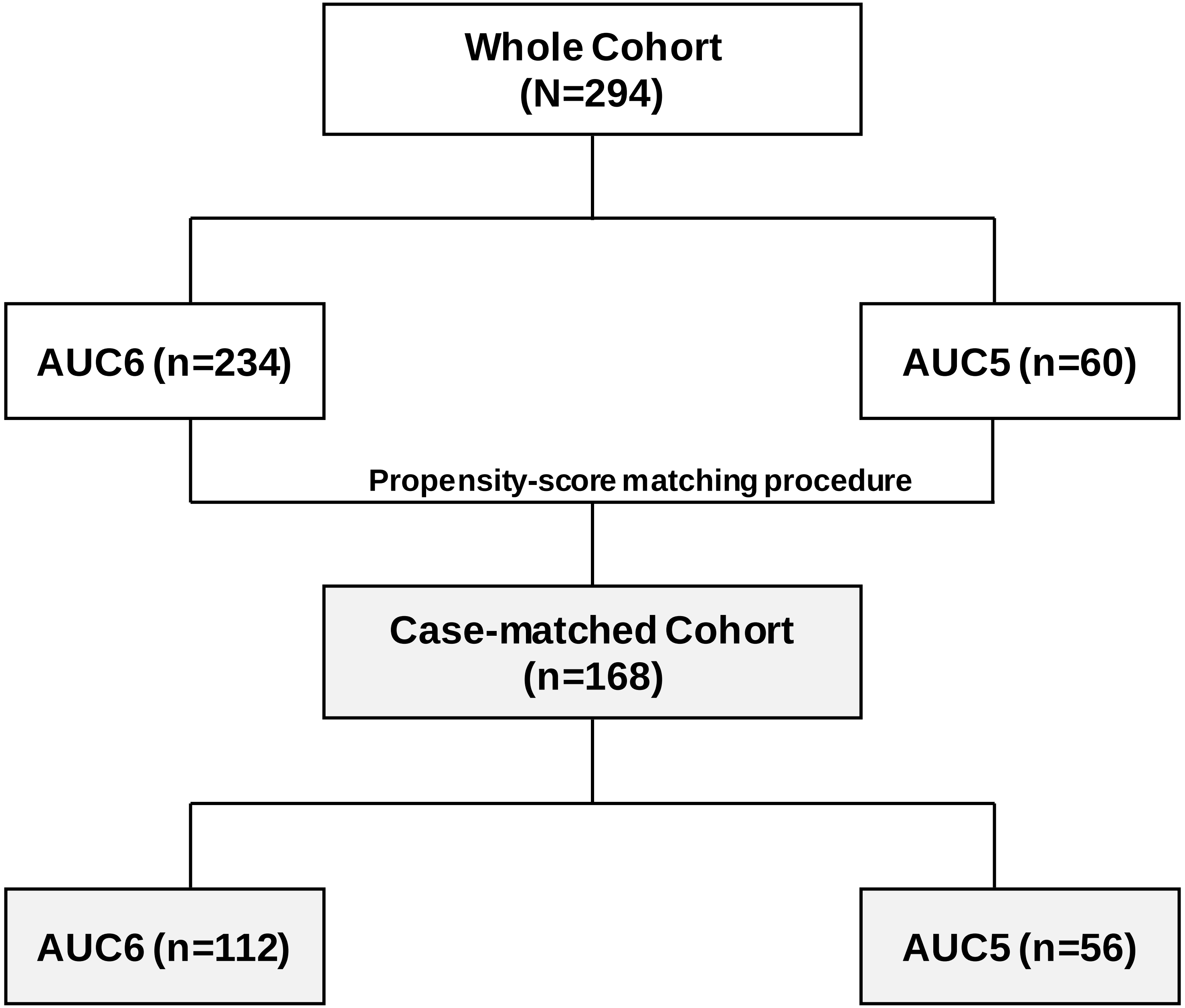

Supplementary Fig 2. Serial platelet (Plt) levels during neoadjuvant docetaxel/carboplatin/trastuzumab/pertuzumab treatment according to carboplatin dose. Serial Plt levels according to carboplatin dose were compared using two-way analysis of variance tests in the (A) whole cohort (n=294) and (B) matched cohort (n=168) (*p*=0.010 and *p*=0.283, respectively).

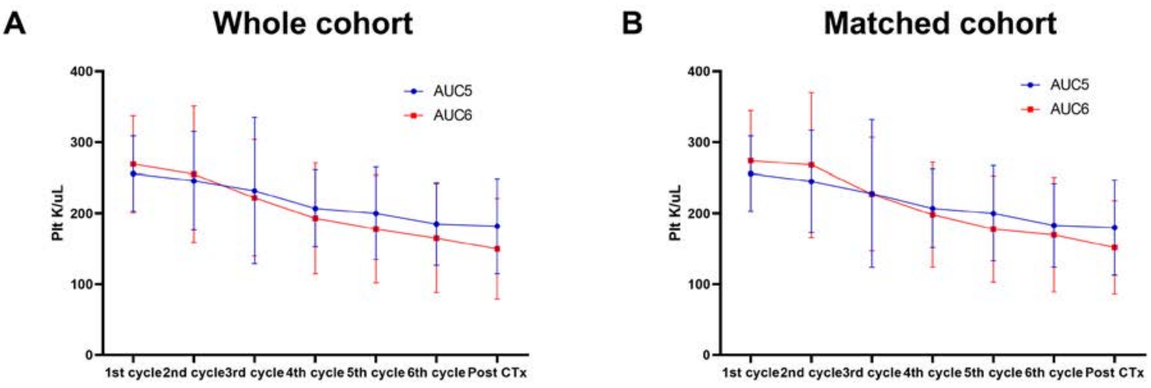

Supplement: Supplementary file 1 — Appendix S1 [file CAM4-12-1409-s001.pdf]
